# Expert opinion in the management of aqueous Deficient Dry Eye Disease (DED)

**DOI:** 10.1186/s12886-015-0122-z

**Published:** 2015-10-13

**Authors:** Aileen Sy, Kieran S. O’Brien, Margaret P. Liu, Puja A. Cuddapah, Nisha R. Acharya, Thomas M. Lietman, Jennifer Rose-Nussbaumer

**Affiliations:** Department of Ophthalmology, California Pacific Medical Center, San Francisco, CA USA; F.I. Proctor Foundation, University of California, Room S309, 513 Parnassus Ave, UCSF, San Francisco, CA 94143-0412 USA; Department of Ophthalmology, University of California, San Francisco, CA USA

**Keywords:** Dry eye disease, Keratoconjunctivitis sicca, Sjögrens

## Abstract

**Background:**

Dry eye disease (DED) affects millions of people worldwide. There are a variety of new treatments beyond traditional therapies such as preservative free artificial tears. Here, we conduct a survey to identify the most common treatments used among specialists and assess their interest in newer therapies.

**Methods:**

An international survey was distributed to dry eye researchers and expert practitioners via an internet survey. The survey data collected were analyzed with descriptive statistics.

**Results:**

One hundred and fifteen respondents completed the survey; of these, 66 % were cornea specialists. The most commonly prescribed topical treatments included cyclosporine A (CSA) 0.05 % (71/104, 68 %), fluorometholone (FML) 0.1 % (59/99, 60 %), loteprednol etabonate 0.5 % (50/99, 51 %), and autologous serum eye drops (ASD; 48/97, 49 %). The most commonly prescribed non-topical medications included essential fatty acid supplements (72/104, 69 %), low-dose doxycycline (oral; 61/100, 61 %), and flaxseed supplements (32/96, 33 %) as well as punctal plugs (76/102, 75 %). Respondents reported treatment with topical corticosteroids for 2 to 8 weeks (46/86, 53 %), followed by less than 2 weeks (24/86, 28 %) and with topical CSA between 2 to 8 weeks (45/85, 53 %) followed by 2 to 6 months (24/85, 28 %). The top three signs and symptoms reported to indicate treatment response were, in order, fluorescein staining of the cornea, reduction in foreign body sensation, and reduction in burning sensation.

**Conclusion:**

This survey offers insight into current expert opinion in the treatment of DED. The results of this survey are hypothesis generating and will aid in the design of future clinical studies.

## Background

DED results from reduced tear production or excessive tear evaporation and is estimated to affect 1,000 000 to 4.9,000 000 people in the United States, primarily in elderly and female populations [1]. Symptoms of burning and tearing can cause a significant reduction in quality of life for patients. In severe cases, they may develop complications such as corneal scarring, bacterial keratitis, and vision loss [1, 2].

DED is divided by etiology into two categories: aqueous deficient (keratoconjunctivitis sicca) and evaporative disorders. Treatment of aqueous deficient DED has traditionally started with artificial tears and topical lubricants [3]. Topical anti-inflammatory medications, including corticosteroids and cyclosporine A 0.05 %, are commonly used for more moderate to severe cases [3]. Although not fully understood, ocular surface damage from either disorder may incite an inflammatory response that further worsens DED [4]. This has inspired many studies exploring new anti-inflammatory treatments for both aqueous deficient and evaporative DED, including topical tacrolimus, topical autologous serum, oral tetracyclines and omega fatty acid supplements [5–8].

In this study, we conduct an international survey of dry eye experts to identify the most common treatments used for aqueous deficient DED beyond traditional therapies such as preservative free artificial tears (PFATs).

## Methods

A survey was distributed to researchers and expert practitioners in the field of DED via the internet survey tool SurveyMonkey (surveymonkey.com, LLC; Palo Alto, California) in April 2013. Experts were identified by collecting corresponding author information from recently published manuscripts in the field of dry eye. PubMed and Web of Science were queried using the search terms “sjögren eye,” “keratoconjunctivitis sicca,” and “dry eye,” and all articles published from November 2008 to November 2012 were included. Those who had previously opted out of receiving emails from SurveyMonkey or those with invalid email addresses were pre-determined to be ineligible. The survey was anonymous and no participant identifiers of name, gender or age were collected; all recipients had the option of participating or opting out. IRB exemption was obtained from the University of California, San Francisco Committee on Human Research. The study adhered to the Declaration of Helsinki and all federal and state laws. After its initial distribution in April 2013, two survey reminders were sent and the survey was closed on July 1, 2013.

The survey consisted of 11 questions (Appendix 1), which were developed after a literature search identified the most common available medications for DED. Survey recipients were first presented with a clinical scenario of a patient with symptoms and physical exam findings consistent with aqueous deficient DED. Respondents were then asked to describe their use of various therapies for this patient including punctal plugs, topical, and oral medications using Likert scales. Next, participants were asked to indicate which of these therapies they would be interested in using more and primary limitations to their current use. Respondents were questioned regarding their routine treatment algorithms and treatment milestones they use to guide therapy. Lastly, general demographic questions were asked. Descriptive statistical analyses were performed using Stata 10.0 (StataCorp, College Station, TX).

## Results

A literature search identified 1450 unique email addresses. Of these, 111 email addresses had previously opted out of SurveyMonkey; subsequently, the survey was sent to 1339 recipients. A total of 115 (8.6 %) recipients completed the survey. The majority identified themselves as cornea specialists (66 %); the remaining respondents were comprehensive ophthalmologists (16 %), non-clinical researchers (6 %), optometrists (6 %) and other (6 %). Most respondents reported treating over 60 aqueous deficient dry eye patients in the last year (76 %). For clinicians reporting duration of practice, the average number of years in clinical practice was 16.5 years (median 13, range 3–40 years). Respondents were primarily from Europe (35 %) and the United States/Canada (28 %); others were from Asia/Indian subcontinent, Central and South America, Australia/New Zealand, the Middle East and Africa.

The most commonly prescribed topical treatments (defined as responses of use always, frequently or sometimes) for aqueous deficient DED include cyclosporine A (CSA) 0.05 % (71/104, 68 %), fluorometholone (FML) 0.1 % (59/99, 60 %), loteprednol etabonate 0.5 % (50/99, 51 %), and autologous serum eye drops (ASD; 48/97, 49 %) (Table 1). Respondents reported improvement with CSA treatment between 2 weeks and 2 months (45/85, 53 %) followed by 2 to 6 months (24/85, 28 %). Only 2 respondents indicated improvements with cyclosporine treatment at 6 months and longer. When asked how long respondents treated patients with topical corticosteroids (including the taper period), most reported 2 to 8 weeks (46/86, 53 %), followed by less than 2 weeks (24/86, 28 %).

**Table 1:** Respondents’ use of various topical medications for patients with clinical scenario consistent with aqueous deficient DED (artificial tears excluded)

Topical Medications	Never use	Rarely use	Sometimes	Frequently	Always use
	*N* (%)	*N* (%)	*N* (%)	*N* (%)	*N* (%)
Cyclosporine A, 0.05 %, ophthalmic emulsion (RESTASIS) (*N* = 105)	22 (21)	12 (11)	21 (20)	35 (33)	15 (14)
Cyclosporine A, 0.1 %, ophthalmic emulsion (*N* = 93)	49 (53)	22 (24)	15 (16)	6 (6)	1 (1)
diclofenac sodium 0.1 % (*N* = 97)	63 (65)	21 (22)	12 (12)	1 (1)	0 (0)
prednisolone acetate 1 % (*N* = 96)	39 (41)	28 (29)	20 (21)	9 (9)	0 (0)
fluorometholone 0.1 % (*N* = 99)	22 (22)	18 (18)	34 (35)	23 (23)	2 (2)
Loteprednol etabonate 0.5 % (*N* = 99)	28 (28)	21 (21)	18 (18)	29 (30)	3 (3)
Resolvin E1 (omega −3 FA) eye drop (*N* = 95)	64 (67)	7 (8)	20 (21)	4 (4)	0 (0)
Diquafosol 3 % ophthalmic solution (*N* = 93)	75 (81)	10 (11)	5 (5)	3 (3)	0 (0)
Tacrolimus 0.03 % ophthalmic emulsion (*N* = 94)	60 (64)	25 (27)	7 (7)	1 (1)	1 (1)
Autologous serum eye drops (*N* = 97)	30 (31)	19 (19.5)	27 (28)	19 (19.5)	2 (2)
Vitamin A eye drops (0.01 % all trans-retinoic acid) (*N* = 96)	39 (41)	27 (28)	22 (23)	7 (7)	1 (1)

The most commonly prescribed oral medications (defined as use always, frequently or sometimes) included essential fatty acid supplements (72/104, 69 %), low-dose doxycycline (oral; 61/100, 61 %), and flaxseed supplements (32/96, 33 %) (Table 2). Punctal plugs were commonly used as well (76/102, 75 %; Table 2).

**Table 2:** Respondents’ use of various non-topical medications for patient with clinical scenario consistent with aqueous deficient DED

Non-topical medications	Never use	Rarely use	Sometimes	Frequently	Always use
	*N* (%)	*N* (%)	*N* (%)	*N* (%)	*N* (%)
Essential Fatty Acids (omega-3 fatty acid, linolenic acid) supplements (*N* = 104)	15 (14)	17 (16)	29 (28)	31 (30)	12 (12)
Flaxseed supplements (*N* = 96)	42 (44)	22 (23)	14 (14)	15 (16)	3 (2)
Doxycycline, low dose, oral (*N* = 100)	18 (18)	21 (21)	35 (35)	25 (25)	1 (1)
Pilocarpine, oral (*N* = 98)	62 (63)	24 (25)	7 (7)	3 (3)	2 (2)
Cevimeline, oral (*N* = 96)	72 (75)	16 (17)	5 (5)	2 (2)	1 (1)
Hydroxychloroquine 6-7 mg/kg daily (*N* = 97)	74 (76)	17 (18)	5 (5)	1 (1)	0 (0)
Rituximab (*N* = 97)	82 (85)	13 (13)	2 (2)	0 (0)	0 (0)
Cyclosporine, oral (*N* = 99)	82 (83)	14 (14)	2 (2)	1 (1)	0 (0)
Corticosteroids, oral (*N* = 98)	72 (73)	21 (21)	3 (3)	2 (2)	0 (0)
Dehydroepiandrosterone (DHEA), oral (*N* = 98)	85 (87)	9 (9)	3 (3)	0 (0)	1 (1)
Punctal Plugging (*N* = 102)	10 (10)	16 (16)	39 (38)	33 (32)	4 (4)

Respondents reported wanting to prescribe cyclosporine A 0.05 % (52/79, 66 %), autologous serum eye drops (39/73, 53 %), resolvin E1 (omega 3 fatty acid) eye drops (31/72, 43 %), and diquafosol 3 % (31/75, 41 %) more often. Of those who indicated they would use cyclosporine A 0.05 % and autologous serum eye drops more, the most cited reason for not being able to prescribe these medications currently was high cost or lack of insurance coverage. The primary reason for not being able to prescribe resolvin E1 eye drops and diquafosol 3 % was difficulty obtaining the medication, although respondents also indicated an interest in more research supporting the use of resolvin E1 drops. There was also moderate interest in cyclosporine A 1 % (28/72, 39 %), vitamin A eye drops 0.01 % (30/77, 39 %), and tacrolimus 0.03 % (25/73, 34 %). The most cited reasons for not being able to prescribe these medications was availability of cyclosporine A 1 % and vitamin A eye drops, and insufficient data to support tacrolimus use.

Regarding alternatives to topical therapy, study participants indicated they would be most interested in using essential fatty acids (EFA) (34/77, 44 %), punctal plugs (31/77, 40 %) and low-dose doxycycline (25/69, 36 %). About half of the participants cited cost as a limiting factor for use of EFA, while the other half cited insufficient research data to support its use. Cost was also the primary prohibitive factor for punctal plugs. For those interested in using doxycycline, respondents listed “other” reasons as the limiting factor. Participants also expressed interest in more investigation into essential fatty acids, rituximab and dehydroepiandrosterone (DHEA).

The top three signs and symptoms reported to indicate treatment response were, in order, fluorescein staining of the cornea, reduction in foreign body sensation, and reduction in burning sensation, together accounting for over 53 % of the responses (134/255). The remaining outcomes were, in order: tear break-up time, improved tear production, lissamine green staining, Schirmer type 1 (no anesthetic), and rose-bengal staining.

## Discussion

The goal of this survey was to characterize the most commonly prescribed treatments for aqueous deficient DED among dry eye specialists and to identify therapeutic options that may be of interest for future study. Our results indicate that while many ophthalmologists are commonly using topical therapies such as steroids and cyclosporine A 0.05 %, they are also turning to oral and other non-topical therapies.

Among topical therapies for aqueous-deficient DED, respondents were most commonly using topical steroids (fluorometholone 0.1 % and loteprednol etabonate 0.5 %), CSA 0.05 %, and autologous serum eye drops. While these practice patterns may reflect respondent experience and interpretation of clinical data, they are also likely affected by local drug availability. The role of inflammation in DED makes topical corticosteroids a natural candidate for therapy, but significant side effects with prolonged corticosteroid therapy, including secondary glaucoma, infection and cataract formation, have limited their use. One randomized controlled trial found loteprednol etabonate 0.5 % to be effective over placebo in patients with moderate clinical inflammation [9]. Topical CSA is an attractive alternative given its relative safety. In addition, it is the only medication approved by the US Food and Drug Administration for moderate to severe DED, and has been well studied in clinical trials [10–12]. These clinical trials also studied the use of CSA 0.1 %, and many found it to be as efficacious as the 0.05 % formulation as well as safe in both short and long term studies [11, 12]. Respondents in this survey demonstrated an interest in using a stronger formulation of CSA (0.1 %), but are prohibited by its lack of availability. Compounding pharmacies are required to obtain CSA 0.1 % as commercial formulation is undergoing regulatory review in Europe. The optimal duration of therapy with topical CSA is unclear. One study has suggested that after a year of standard twice daily CSA 0.05 % therapy, patients can be successfully weaned to once daily therapy without significant worsening of their symptoms. [13] Respondents of this survey reported most commonly seeing improvements with CSA therapy in shorter time periods (2 weeks to 2 months). ASD are thought to alleviate dry eye by supplying tear components such as various growth factors and vitamins that aid in the maintenance of the ocular surface; it has been studied in a handful of small clinical studies, the majority of which have found it to be more effective than artificial tears [7, 14–17]. However, there have not been studies comparing ASD to other medications such as CSA. While CSA, topical corticosteroid and ASD appear to have been accepted into the community practice, there is a lack of comparative studies between these agents.

Among non-topical therapies for DED patients, respondents were most commonly using punctal plugs, oral EFA supplements, low-dose oral doxycycline and flaxseed supplements. Punctal plugs have been studied in multiple clinical trials, and a recent Cochrane review found that although the literature did not decisively support its efficacy for dry eye treatment, the data did suggest “usefulness” in DED [18]. EFA supplements are traditionally used to treat evaporative DED or meibomian gland disease. Systemic EFA supplements have been studied in small- to moderate-sized clinical trials; only a few showed improvements for limited measures of DED, with others finding no statistically significant improvement [19–22]. Doxycycline, an antibacterial with anti-inflammatory effects, gained traction as a therapy for DED when inflammatory pathways were added to the understanding of dry eye pathogenesis [5]. It has primarily been studied in ocular rosacea, but a recent study in a small number of patients suggests doxycycline to be effective in restoring tear film stability in dry eye patients [23–26]. Despite limited data for both omega fatty acids and doxycycline, both are being used by survey respondents, with significant interest in increasing their use.

Study participants reported significant interest in new topical therapies, particularly diquafosol and vitamin A eye drops. Diquafosol (1–3 %), a P2Y2 receptor agonist thought to promote non-glandular secretion of fluid, is available primarily in East Asian countries due to its approval for use in Japan, but is still undergoing clinical review in the United States. Survey respondents from the Asian/Indian subcontinent did not demonstrate a higher frequency of diquafosol use. Diquafosol has been found in a number of prospective clinical trials to be effective compared with placebo and sodium hyaluronate; there have been no studies comparing its efficacy to CSA 0.05 % [27–30]. Vitamin A has been identified in natural tears and vitamin A deficiency is thought to cause evaporative DED. Vitamin A drops have been shown in one moderate-sized trial to significantly improve dry eye signs and symptoms over artificial tears, and had similar efficacy to CSA 0.05 % [31]. Respondents also demonstrated interest in two lesser studied medications: topical tacrolimus and resolvin E1 (EFA) eye drops, and appropriately called for more clinical investigations into these medications. Tacrolimus 0.03 %, a macrolide with immunomodulatory properties, was found in small prospective case series to significantly improve signs of dry eye in Sjogren’s syndrome patients and in graft versus host disease patients refractory to topical CSA 0.05 %; it has yet to be studied in a randomized controlled trial setting [6, 32]. A stronger formulation of tacrolimus (0.1 %) has been approved in Japan and is readily available in East Asian countries, though survey respondents from Asia/Indian sub-continent did not report using this treatment more frequently. Resolvin and other topical EFA formulations have only been studied in animal models [33, 34]. While lack of availability was a significant barrier to respondents using all 4 topical therapies, use of vitamin A drops, tacrolimus and topical EFA was also limited by lack of clinical data.

Our results suggest non-topical therapies, such as rituximab and DHEA, are of great interest to clinicians as targets for future research in the treatment of aqueous deficient DED. Rituximab has been reported in case reports for keratoconjunctivitis sicca, but has mostly been studied in clinical trials for primary Sjogren’s. Results are pending from two recent clinical trials, the Tolerance and Efficacy of Rituximab in Sjogren’s Disease trial and the Trial of Anti-B-Cell Therapy in Patients With Primary Sjögren’s Syndrome [35–39]. Minimal literature exists for the use of sex steroids, such as DHEA, in DED. DHEA supplementation has been studied in Sjogren’s syndrome and has not had promising results [40, 41]. Interestingly, respondents were not interested in cholinerigic agonists such as pilocarpine and cevimeline which have been shown in clinical trials to be effective for dry eye secondary to Sjogren’s syndrome; there was also little interest in hydroxychloroquine, oral cyclosporine or oral steroids [8].

One issue in studying DED is the lack of consensus regarding measures of efficacy in clinical trials. This is compounded by the fact that severity of symptoms do not correlate with exam findings [42]. Outcomes of interest to clinicians in this survey were fluorescein staining of the cornea, reduction in foreign body sensation and reduction in burning sensation. Interestingly, Schirmer type 1 test was not among the top responses despite commonly being used clinically and in clinical trials [42]. The results of this survey may guide the selection of outcomes in future clinical trials.

Limitations of this study include those intrinsic to all surveys, including recall bias and non-response bias. The response rate of 8.6 % is not uncommon and is better than previous dry eye surveys [43, 44]. A strength of the survey is the high proportion of respondents who were corneal specialists, as this is likely to be the population most interested in the topic. Our method of surveying biases toward academic practitioners, and therefore likely represents expert opinion rather than current practices in general. Since the survey did not include traditional therapies such as PFATs, this increased the responses to other therapies such as ASD, which likely does not reflect current practice outside of academic centers. However, this survey is informative about the implementation of newer treatments in DED and is hypothesis generating for future areas of study. The survey could not address all possible potential therapies, which may have prohibited the identification of other treatments of interest. Respondents were given the option to suggest additional therapies, but responses were minimal. Finally, as respondents practice patterns are likely limited by drug availability and local treatment guidelines, the geographic diversity of respondents is a strength of the survey.

## Conclusions

The need for alternative, more effective DED treatments is clear. By determining current practice patterns, this survey offers insight into alternative therapies that are already being used amongst practitioners. Additionally, the results of this survey identify therapies of interest and important clinical outcomes that can aid in the development of future clinical studies.

## Consent

Informed consent was obtained from all study participants for the publication of this report.

## Abbreviations

DED: Dry eye disease
CSA: Cyclosporine A
FML: Fluorometholone
ASD: Autologous serum drops
PFATs: Preservative free artificial tears
EFA: Essential fatty acids
DHEA: Dehydroepiandrosterone
